# Human Group A Streptococci Virulence Genes in Bovine Group C Streptococci

**DOI:** 10.3201/eid1601.090632

**Published:** 2010-01

**Authors:** Márcia G. Rato, Ricardo Bexiga, Sandro F. Nunes, Cristina L. Vilela, Ilda Santos-Sanches

**Affiliations:** Universidade Nova de Lisboa, Caparica, Portugal (M.G. Rato, I. Santos-Sanches); Universidade Técnica de Lisboa, Lisbon, Portugal (R. Bexiga, S.F. Nunes, C.L. Vilela); Cambridge University, Cambridge, UK (S.F. Nunes)

**Keywords:** Bovine mastitis, lysogeny, prophages, superantigens, Streptococcus pyogenes, Streptococcus dysgalactiae subsp. dysgalactiae, bacteria, viruses, dispatch

## Abstract

Phage-encoded virulence genes of group A streptococci were detected in 10 (55.6%) of 18 isolates of group C streptococci that had caused bovine mastitis. Bovine isolates carried other genetic determinants, such as composite transposon Tn*1207.3*/Φ10394.4 (100%) and antimicrobial drug resistance genes *erm*(B)/*erm*(A) (22.2%), *lin*B (16.6%), and *tet*(M)/*tet*(O) (66.7%), located on mobile elements.

Strains of *Streptococcus dysgalactiae* subsp. *dysgalactiae* are described as α-hemolytic or nonhemolytic (Lancefield group C) and associated only with animal infections (bovine mastitis), a disease with major economic consequences for the dairy industry (*1*). Group A streptococci (GAS)–specific phage-associated virulence determinants encoding pyrogenic exotoxins or superantigens (*speM*, *ssa*), which are strongly associated with severe diseases such as scarlet fever, streptococcal toxic shock syndrome, and rheumatic fever, have been described among human group C streptococci (GCS) or group G streptococci (GGS) (*S*. *dysgalactiae* subsp. *equisimilis*) (*2*) but not among α-hemolytic GCS (*S*. *dysgalactiae* subsp. *dysgalactiae*) of bovine origin. In contrast, M protein or M-like proteins were found in human GGS/GCS (*S*. *dysgalactiae* subsp. *equisimilis*) and in animal GCS (*S*. *dysgalactiae* subsp. *dysgalactiae*) but only in β-hemolytic strains (*3*).

Composite transposons and other genetic determinants also considered to be located in specific mobile elements such as macrolide (either encoding methylases [*erm* genes] or efflux pumps [*mef* genes]) and tetracycline resistance determinants (*tet* genes) have been found among streptococcal species of human origin. We studied a collection of field isolates of bovine GCS *S*. *dysgalactiae* subsp. *dysgalactiae* to search for genetic determinants, particularly those carried by mobile elements known to be transferred among human GAS and GGS/GCS.

## The Study

We studied 18 α-hemolytic *S*. *dysgalactiae* subsp. *dysgalactiae* field isolates of Lancefield group C that had caused bovine subclinical mastitis. Isolates were obtained from 304 milk samples of 248 cows from 8 farms in Portugal that were included in the study. Detailed information regarding isolation methods and identification of field isolates by biochemical methods was described in a study of the subclinical mastitis–associated pathogen *S*. *uberis* (*4*). To confirm identification of *S*. *dysgalactiae* subsp. *dysgalactiae*, the 16S rRNA gene was amplified by PCR and sequenced (*5*). *Sma*I/*cfr*9I-digested DNA banding patterns were obtained by pulsed-field gel electrophoresis for clone identification as described (*4*).

All genes analyzed by PCR are shown in the Appendix Table. The *emm* gene subtyping was performed as described (www.cdc.gov/ncidod/biotech/strep/M-ProteinGene_typing.htm). Primers used and conditions for PCR were essentially as described elsewhere (Appendix Table).

Samples without DNA and strains lacking (negative) or carrying (positive) specific genes were used as controls in the PCR. Results were consistent in 2 or 3 PCRs that included these controls. Sequencing of all virulence gene amplicons was performed with the same primers used for amplification (STAB-Vida, Lisbon, Portugal). All sequences were compared with sequences in GenBank by using the BLAST alignment tool (www.ncbi.nlm.nih.gov/BLAST).

Antimicrobial drug resistance against macrolides (erythromycin), lincosamides (pirlimycin), and tetracycline was determined as described (*10*). Macrolide resistance phenotypes identified were M (resistance to macrolides) and MLS_B_ (resistance to macrolides, lincosamides and streptogramins B).

We detected bacteriophage-associated virulence genes *spe*M, *spe*K, *spe*C, *spd*1, and *spe*L. Overall, *spe*M was found in 10 (55.6%) of 18 bovine GCS isolates, *spe*K in 9 (50%), *spe*C and *spd*1 in 6 (33%), and *spe*L in 4 (22.2%). All but 1 of the PCR products showed expected sizes (Appendix Table). Tn*1207*.*3*/Φ10394.4 composite transposon left junction amplicon showed a size of 380 bp instead of 453–6,807 bp as described for GAS (*9*). No amplification was observed for the right junction of this genetic element.

**Figure 1:**
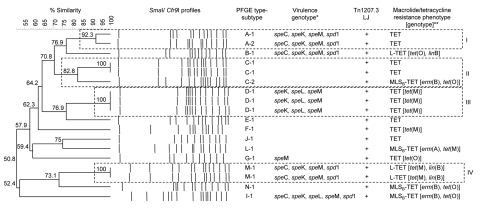
Dendrogram and pulsed-field gel electrophoresis (PFGE) profiles of group C streptococci (*Streptococcus dysgalactiae* subsp. *dysgalactiae*) subclinical mastitis isolates from 8 dairy herds, Portugal. PFGE type-subtype, virulence genotype, antimicrobial drug resistance phenotypes, and genotypes of each isolate are indicated. The dendrogram was produced by using Dice coefficients and unweighted pair group method using arithmetic averages. Default clustering settings of 0.00% optimization (i.e., the relative distance an entire lane is allowed to shift in matching attempts) and 1.5% band position tolerance were used. *All isolates were negative for *spe*A, *ssa*, *spe*H, *spe*J, *spe*I, and *sla*A genes and for Tn*1207.3*/Φ10394.4 element right junction tested by PCR; **All isolates were negative for *mef*A, *tet*(T), *tet*(W), *tet*(L), *tet*(Q), *tet*(S) and *tet*(K) genes tested by PCR; TET, resistance only to tetracycline; MLS_B_-TET, resistance to macrolides, lincosamides, streptogramin B, and TET; L-TET, susceptibility to macrolides and resistance to lincosamides (L phenotype) and TET; Tn1207.3 LJ, Tn*1207.3*/Φ10394.4 element left junction. Clusters are shown in roman numerals on the right.

The *emm* gene encoding the antiphagocytic M surface protein was not amplified in any of the 18 bovine GCS isolates; therefore, no *emm* types were obtained. Subsets of isolates were erythromycin and pirlimycin resistant (MLS_B_ phenotype) and contained *erm*(B) or *erm*(A) genes (22.2%) or erythromycin susceptible and pirlimycin resistant and contained the *linB* gene (16.6%). All isolates were tetracycline resistant with a subset (66.7%) carrying *tet*(M) or *tet*(O) tetracycline resistance determinants. Distribution of bacteriophage-associated virulence genes and other characteristics of strains are shown in Figure 1.

**Figure 2:**
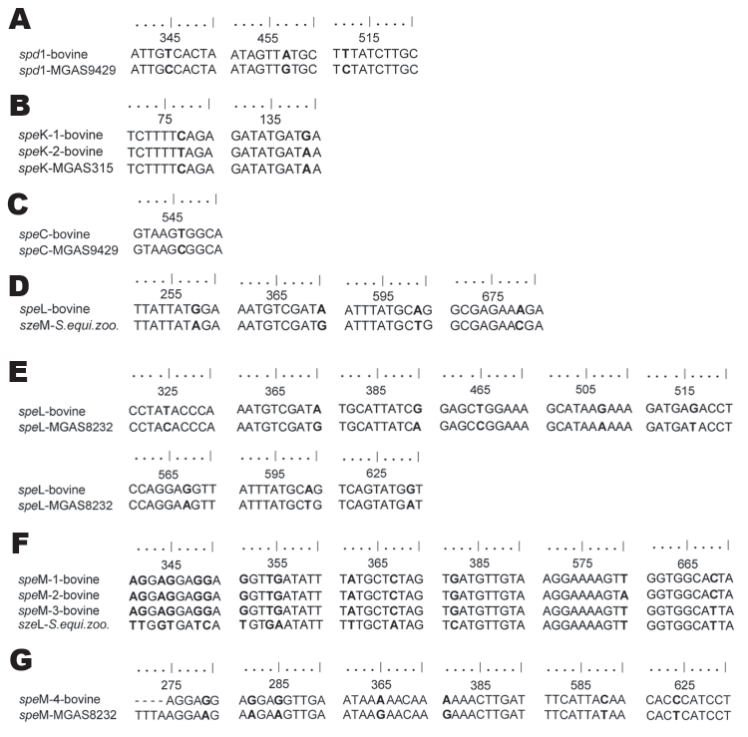
Alignments of bovine group C streptococci (*Streptococcus dysgalactiae* subsp. *dysgalactiae*) alleles of virulence genes from 8 dairy herds, Portugal, with sequences from the National Center for Biotechnology (Bethesda, MD, USA) database showing base differences between sequences. The alignments were created by using BioEdit sequence alignment editor (www.mbio.ncsu.edu/BioEdit/bioedit.html). Nucleotide differences are shown in **boldface**. A) *spd*1 (99% maximum identity); B) *spe*K (99% maximum identity); C) *spe*C (99% maximum identity); D) *spe*L–*sze*M (99% maximum identity); E) *spe*L (97% maximum identity; F) *spe*M alleles 1, 2, and 3–*sze*L (98%–99% maximum identity); G) *spe*M allele 4 (98% maximum identity). *S*. *equi*. *zoo*., *S*. *equi* subsp. *zooepidemicus*.

Sequences of all virulence genes were compared by using the BioEdit sequence alignment editor (www.mbio.ncsu.edu/BioEdit/bioedit.html). One different allele was found for each of the following gene sequences: *spd*1 (among 6 strains), *spe*C (among 6 strains), and *spe*L (among 4 strains). Two alleles were found for *spe*K (among 9 strains) (*spe*K-1 and *spe*K-2), and 4 alleles were found for *spe*M gene sequences (among 10 strains) (*spe*M-1, *spe*M-2, *spe*M-3, and *spe*M-4). Bovine alleles had sizes of 386 bp (*spd*1), 222 bp (*spe*C), 444 bp (*spe*L), 232 bp (*spe*K), and 357 bp (*spe*M). Examples of alignments between bovine virulence gene alleles with sequences from GenBank (only most similar ones) are shown in Figure 2.

## Conclusions

Using PCR, we determined that bovine GCS *S*. *dysgalactiae* subsp. *dysgalactiae* strains (55.6%) carried >1 GAS-specific bacteriophage virulence-associated genes (*spd*1, *spe*C, *spe*K, *spe*L, and *spe*M). This finding suggested that bacteriophages may also play a role in the genetic plasticity and virulence of animal GCS.

The *spe*L allele from bovine strains showed higher similarity with the *sze*M allele (99% maximum identity) from *S*. *equi* subsp. *zooepidemicus* than with the *spe*L allele (97% maximum identity) from *S*. *pyogenes*. The *sze*M gene encodes a superantigen in *S*. *equi* subsp. *zooepidemicus*, which is primarily a pathogen of nonhuman animal species. This organism causes mastitis in cows and mares and is most frequently found in horses (*14*). We also observed that 3 of the *spe*M alleles found among bovine strains (*spe*M-1, *spe*M-2, and *spe*M-3) also showed higher similarity with superantigen-encoding gene *sze*L from *S*. *equi* subsp. *zooepidemicus* than with *spe*M gene sequence from *S*. *pyogenes*. Another allele (*spe*M-4) showed higher similarity with the *sdm* gene from *S*. *dysgalactiae* subsp. *dysgalactiae* than with the speM gene from *S*. *pyogenes*.

The remaining alleles (*spd*1, *spe*C, *spe*K-1, and *spe*K-2) from the GCS *S*. *dysgalactiae* subsp. *dysgalactiae* bovine strains showed high similarity with *S*. *pyogenes* superantigen genes (98%–99% maximum identity). This finding supports our hypothesis that GAS prophages may play a role in the genetic plasticity of this pathogen. The *spe*C and *spd*1 genes are known to be localized on the same GAS prophage (*15*), and both genes were detected in 6 bovine GCS *S*. *dysgalactiae* subsp. *dysgalactiae* isolates in our study.

None of 18 α-hemolytic group C *S*. *dysglacatiae* subsp. *dysgalactiae* bovine isolates in this study were typed by *emm*-typing because amplification products in the PCR specific for the M surface protein gene *emm* were not obtained. This result is consistent with those of a report that β-hemolytic, but not α-hemolytic, group C *S*. *dysglacatiae* subsp. *dysgalactiae* isolates of animal origin contained the *emm* gene (*3*).

Amplification (380-bp product) of the left junction of the composite transposon in bovine isolates suggests that this mobile element may be inserted in a similar location, the *comEC* locus, as mapped in *S*. *pyogenes* and *S*. *dysglactiae* subsp. *equisimilis*. Absence or unexpected PCR products specific for any of the junctions of this element have been reported in other studies and attributed to possible lack of homology between the target and primers used (*9*). Detection of the *linB* gene carried by a large conjugative plasmid (*13*) in 3 of 18 bovine GCS *S*. *dysgalactiae* subsp. *dysgalactiae* isolates is indicative of horizontal gene transfer.

Our findings indicate that α-hemolytic bovine GCS isolates, which are known to be environmental or contagious pathogens and a cause of bovine mastitis, may be reservoirs of virulence genes encoded by prophages of human-specific GAS. These genes encode exotoxins, superantigens, and streptodornases, which are responsible for GAS virulence and pathogenesis, and may be transferred to other streptococci of human origin by horizontal genetic transfer. Therefore, α-hemolytic isolates should not be disregarded as putative infectious disease agents in humans.

## Supplementary Material

Appendix Table Genes analyzed by PCR and primers used, for group C streptococci, from 8 dairy herds, Portugal
