# Supplementary material for: Human Group A Streptococci Virulence Genes in Bovine Group C Streptococci
Source: Emerg Infect Dis. 2010 Jan;16(1):116–9. doi: 10.3201/eid1601.090632 (PMC2874356; doi:10.3201/eid1601.090632)
Supplement: Appendix Table — Genes analyzed by PCR and primers used, for group C streptococci, from 8 dairy herds, Portugal [file 09-0632_appT-s1.pdf]

Appendix Table. Genes analyzed by PCR and primers used, for group C streptococci, from 8 dairy herds, Portugal

Primer	Sequence (5´ ? 3´)	Expected product, bp	Reference
Prophage-associated virulence determinants			
Pyrogenic exotoxins			
ssa (forward)	GTGTAGAATTGAGGTAATTG	706	(6)
ssa (reverse)	TAATATAGCCTGTCTCGTAC		
*spe*A (forward)	CTTAAGAACCAAGAGATGGC	200	(6)
*spe*A (reverse)	ATAGGCTTTGGATACCATCG		
speC (forward)	CATCTATGGAGGAATTACGC	246	(6)
*spe*C (reverse)	TGTGCCAATTTCGATTCTGC		
*spe*H (forward)	AGATTGGATATCACAGG	416	(6)
*spe*H (reverse)	CTATTCTCTCGTTATTGG		
*spe*I (forward)	AAGGAAAAATAAATGAAGGTCCGCCAT	217	(7)
*spe*I (reverse)	TCGCTTAAAGTAATACCTCCATATGAATTCTTT		
*spe*J (forward)	ATCTTTCATGGGTACG	535	(6)
*spe*J (reverse)	TTTCATGTTTATTGCC		
*spe*K (forward)	TATCGCTTGCTCTATACACTACTGAGAGT	233	(7)
*spe*K (reverse)	CCAAACTGTAGTATTTTCATCCGTATTAAA		
*spe*L (forward)	GGACGCAAGTTATTATGGATGCTCA	460	(7)
*spe*L (reverse)	TTAAATAAGTCAGCACCTTCCTCTTTCTC		
*spe*M (forward)	GCTTTAAGGAGGAGGAGGTTGATATTTATGCTCTA	411	(7)
*spe*M (reverse)	CAAAGTGACTTACTTTACTCATATCAATCGTTTC		
DNase 1			
*spd*1 (forward)	CCCTTCAGGATTGCTGTCAT	400	(8)
*spd*1 (reverse)	ACTGTTGACGCAGCTAGGG		
Phospholipase A2e			
*sla*A (forward)	CTCTAATAGCATCGGCTACGC	440	(8)
*sla*A (reverse)	AATGGAAAATGGCACTGAAAG		
Composite transposon			
Tn*1207.3*/F10394.4 RJ* (forward)	CGAGGAGTTAGTATGGAAAC	473	(9)
Tn*1207.3*/F10394.4 RJ* (reverse)	CCCATAATAGGCAACTGGTCTCCAGC		
Tn*1207.3*/F10394.4 LJ* (forward)	TCTTCGCCGCATAAACCCTATC	453/6,807†	(9)

Tn*1207.3*/F10394.4 LJ* (reverse)	CCTTTGACCAATGAAGTGACCTTT		
Antimicrobial drug resistance determinants			
Macrolide resistance			
mef(A) (forward)	GACCAAAAGCCACAATTGTGGA	1,432	(10)
mef(A) (reverse)	CCTCCTGTCTATAATCGCATG		
erm(A) [subclass *erm*(TR)] (forward)	CCCGAAAAATACGCAAAATTTCAT	590	(10)
erm(A) [subclass *erm*(TR)] (reverse)	CCCTGTTTACCCATTTATAAACG		
erm(B) (forward)	GGAGTGATACATGAACAAAAATA	531	(10)
erm(B) (reverse)	TTCCTTTTAGTAACGTGTAACTTT		
Tetracycline resistance			
tet(M) (forward)	TGGAATTGATTTATCAACGG	1,080	(10)
tet(M) (reverse)	TTCCAACCATACAATCCTTG		
tet(O) (forward)	AACTTAGGCATTCTGGCTCAC	515	(11)
tet(O) (reverse)	TCCCACTGTTCCATATCGTCA		
tet(T) (forward)	AAGGTTTATTATATAAAAGTG	169	(12)
tet(T) (reverse)	AGGTGTATCTATGATATTTAC		
tet(W) (forward)	GAGAGCCTGCTATATGCCAGC	168	(12)
tet(W) (reverse)	GGGCGTATCCACAATGTTAAC		
tet(Q) (forward)	TTATACTTCCTCCGGCATCG	904	(11)
tet(Q) (reverse)	ATCGGTTCGAGAATGTCCAC		
tet(S) (forward)	GAAAGCTTACTATACAGTAGC	169	(12)
tet(S) (reverse)	AGGAGTATCTACAATATTTAC		
tet(L) (forward)	TCGTTAGCGTGCTGTCATTC	267	(11)
tet(L) (reverse)	GTATCCCACCAATGTAGCCG		
tet(K) (forward)	TCGATAGGAACAGCAGTA	169	(11)
tet(K) (reverse)	CAGCAGATCCTACTCCTT		
Lincosamide resistance			
lin(B) (forward)	CCTACCTATTGTTTGTGGAA	925	(13)
lin(B) (reverse)	ATAACGTTACTCTCCTATTC		
M protein			
emm (forward)	TATT(C/G)GCTTAGAAAATTAA	Variable	‡
emm (reverse)	GCAAGTTCTTCAGCTTGTTT		

*RJ, right junction; LJ, left junction.

†The expected ampicon size was 454 bp according to the reported organization of the Tn1207.3 element or 6,807 bp according to the reported sequence of F10394.4 (9).

‡www.cdc.gov/ncidod/biotech/strep/M-ProteinGene_typing.htm
